# Genomics of Avian Viral Infections

**DOI:** 10.3390/genes10100814

**Published:** 2019-10-15

**Authors:** Jacqueline Smith

**Affiliations:** The Roslin Institute, University of Edinburgh, Easter Bush Campus, Midlothian, Scotland EH25 9RG, UK; jacqueline.smith@roslin.ed.ac.uk

**Keywords:** chicken, virus, resistance, avian influenza, Marek’s Disease, Newcastle Disease, poxvirus, interferon, vaccine

## Abstract

The poultry industry currently accounts for the production of around 118 million metric tons of meat and around 74 million metric tons of eggs annually. As the global population continues to increase, so does our reliance on poultry as a food source. It is therefore of vital importance that we safeguard this valuable resource and make the industry as economically competitive as possible. Avian viral infections, however, continue to cost the poultry industry billions of dollars annually. This can be in terms of vaccination costs, loss of birds and decreased production. With a view to improving the health and welfare of commercial birds and to minimizing associated economic losses, it is therefore of great importance that we try to understand the genetic mechanisms underlying host susceptibility and resilience to some of the major viral pathogens that threaten the poultry species. Some avian viruses, through their zoonotic potential, also pose a risk to human health. This Special Issue will present papers that describe our current knowledge on host responses to various viral pathogens, the genetics underlying those responses and how genomics can begin to provide a solution for resolving the threat posed by these infections.

Identifying host genes responsible for resistance to avian viral infections will allow us to understand the mechanisms by which hosts respond to disease and will provide potential targets for marker-assisted selection, improved vaccine design and possible future gene-editing technologies. It is therefore important that we define the host responses in play after viral challenge, whether that be from an infection outbreak or after vaccination. 

This Special Issue brings together a collection of papers focused on genomic analysis of the chicken immune response and associated genetic interactions. Two of the most impactful diseases on the poultry industry are Newcastle Disease and Marek’s Disease. Newcastle Disease Virus (NDV) is a highly contagious paramyxovirus and presents primarily as an acute respiratory disease but with many birds also exhibiting depression, nervous signs and diarrhea. Mortality depends on viral strain but can reach 100% with virulent strains. Marek’s Disease Virus (MDV) is an oncogenic alpha-herpes virus, which is also immunosuppressive. Again, mortality from Marek’s Disease varies depending upon strain, but many birds infected with the virus succumb to secondary infections such as *E.coli* due to the suppression of the immune system. 

Several of the papers presented here focus on these two infections. The response of birds to NDV while under heat stress conditions is thoroughly examined by transcriptomic and proteomic means [1,2,3]. The transcriptomic response of susceptible and resistant birds to Marek’s Disease is also presented [4] along with an analysis of miRNA expression after HVT (Herpesvirus of turkeys) vaccination [5]. A study of chicken serum exosomes after vaccination and challenge, combining both transcriptomics and proteomics is also given [6]. 

Interferons and downstream interferon-stimulated genes (ISGs) play a fundamental role in the host immune system’s fight against a variety of pathogens. Arslan and colleagues [7] describe the effects on interferon lambda regulated genes upon stimulation. ISGs were also induced in chicken embryonic stem cells in the work by Giotis et al. [8], with a view to developing recombinant vaccine vectors. 

As previously mentioned, some avian viruses pose a secondary zoonotic threat – none more so than avian influenza. Understanding the genetics behind resistance not only has obvious utility for the poultry industry, but will be able to inform on human health. The paper by An et al. [9] describes their hypothesis that resistance is mediated by a homeostasis mechanism.

In conclusion, this Issue presents some of the current work in relation to the genomics behind avian viral infections, and brings to the forefront the importance of defining the responses able to mitigate the effects of these diseases.
